# Presumptive thrombotic thrombocytopenic purpura following a hump-nosed viper (*Hypnale hypnale*) bite: a case report

**DOI:** 10.1186/1678-9199-20-26

**Published:** 2014-06-16

**Authors:** Milinda Withana, Chaturaka Rodrigo, Ariaranee Gnanathasan, Lallindra Gooneratne

**Affiliations:** 1 National Hospital, University Medical Unit, Colombo, Sri Lanka; 2 Department of Clinical Medicine, Faculty of Medicine, University of Colombo, 25 Kynsey Road, Colombo 08, Sri Lanka; 3 Department of Pathology, Faculty of Medicine, University of Colombo, Colombo, Sri Lanka

**Keywords:** Thrombotic thrombocytopenic purpura, Hump-nosed viper, Thrombotic microangiopathy, Snakebite, Hemolytic uremic syndrome

## Abstract

Hump-nosed viper bites are frequent in southern India and Sri Lanka. However, the published literature on this snakebite is limited and its venom composition is not well characterized. In this case, we report a patient with thrombotic thrombocytopenic purpura-like syndrome following envenoming which, to the best of our knowledge, has not been reported in the literature before. A 55-year-old woman from southern Sri Lanka presented to the local hospital 12 hours after a hump-nosed viper (*Hypnale hypnale*) bite. Five days later, she developed a syndrome that was characteristic of thrombotic thrombocytopenic purpura with fever, thrombocytopenia, microangiopathic hemolysis, renal impairment and neurological dysfunction in the form of confusion and coma. Her clinical syndrome and relevant laboratory parameters improved after she was treated with therapeutic plasma exchange. We compared our observations on this patient with the current literature and concluded that thrombotic thrombocytopenic purpura is a theoretically plausible yet unreported manifestation of hump-nosed viper bite up to this moment. This study also provides an important message for clinicians to look out for this complication in hump-nosed viper bites since timely treatment can be lifesaving.

## Background

**Figure 1:**
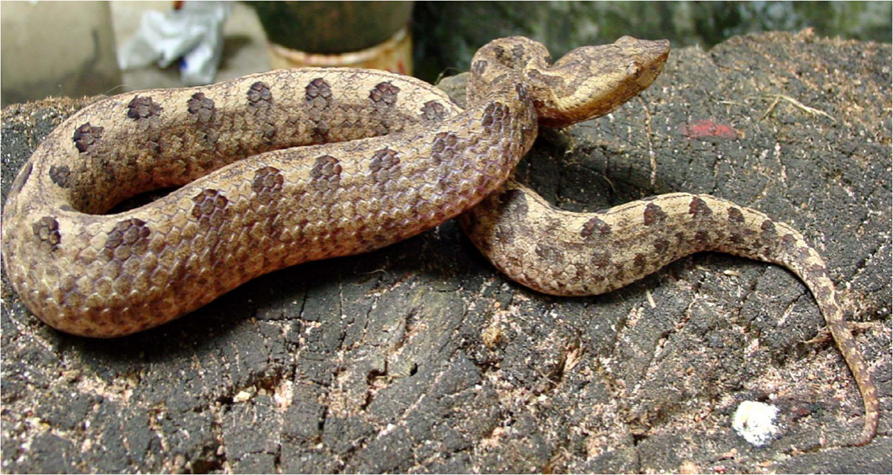
**The hump-nosed viper (*Hypnale hypnale *).**

Hump-nosed vipers of the genus *Hypnale* have three species *Hypnale hypnale* (Figure 1), *Hypnale nepa* and *Hypnale zara*[1]. Their geographical distribution includes mainly Sri Lanka and south western India [2]. Hump-nosed viper bites are the commonest cause of snake envenoming in Sri Lanka accounting for 22 to 77% of all snakebites in different series [3]. Bites are characterized by local inflammation, blistering and tissue necrosis. Systemic envenoming may cause coagulopathy, acute kidney injury and death [1]. Thrombotic microangiopathy (TMA) is one of the proposed pathophysiological mechanisms for its systemic complications [4]. Still, the pathophysiologically related thrombotic thrombocytopenic purpura (TTP) as a complication of snake envenoming has never been reported in the medical literature.

Since TTP has high mortality without prompt recognition and treatment, awareness about this potential complication associated with hump-nosed viper envenoming is lifesaving. Currently there is no licensed antivenom for hump-nosed viper bites in Sri Lanka. The polyspecific antivenom currently available in Sri Lanka is imported from India – which is manufactured against the Indian species of cobra, Russell’s viper, common krait and saw-scaled viper venom – and does not have antibodies against hump-nosed viper venom. Hence, it is ineffective against hump-nosed viper venom and potentially dangerous due to anaphylactic reactions. Therefore, treatment is restricted to supportive care.

## Case presentation

**Figure 2:**
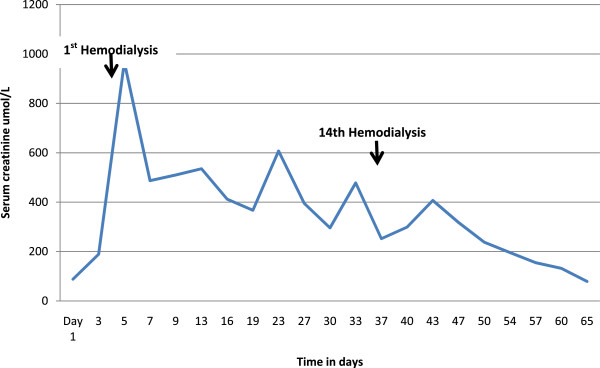
Change in serum creatinine level of the patient during her hospital stay.

**Figure 3:**
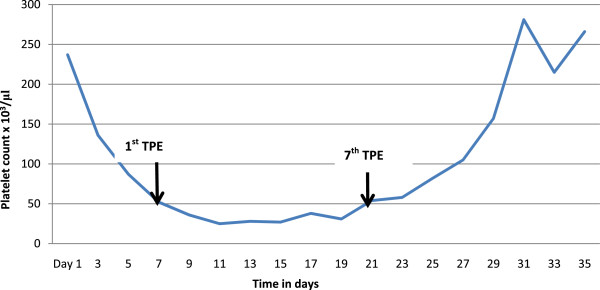
Change in platelet count of the patient during the first 35 days of hospital stay (TPE – therapeutic plasma exchange).

**Figure 4:**
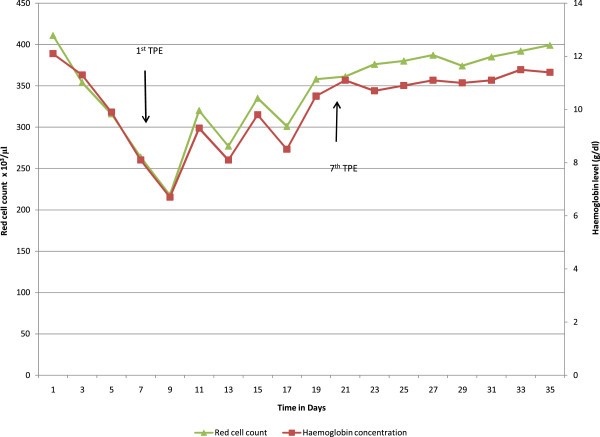
Change in red cell count and hemoglobin during the first 35 days of hospital stay (TPE – therapeutic plasma exchange).

A 55-year-old woman from a rural area of the Southern Province of Sri Lanka presented to the local hospital 12 hours after a snakebite on her left foot. On admission, she had swelling and blistering below the left ankle. The snake was killed and brought to hospital where it was identified by the healthcare staff as a hump-nosed viper (*Hypnale hypnale*) and subsequently confirmed by one of the authors of this study (AG). Her medical history had been unremarkable until this admission and she was not on any long-term medication. The patient was fully conscious, alert and oriented at the admission. There was swelling and blistering on the dorsum of the left foot with two distinct fang marks below the left lateral malleolus. Findings on the rest of her physical examination were unremarkable. A twenty-minute whole blood clotting test (20WBCT) was normal at admission indicating there was no coagulopathy at that point. The initial screening of liver enzymes, prothrombin time and serum creatinine were within reference range.After three days of admission, the patient became unwell with oliguria and serum creatinine began to rise (Figure 2). Hemodialysis was initiated on day 5 (date of snakebite taken as day 0) due to oliguric acute kidney injury. She became confused at this point and deteriorated rapidly within the next 24 hours with reduced level of consciousness. The Glasgow coma scale on day 6 was 7/15 (E2, V2, M3). She had also developed pyrexia at this point with a core temperature of 38.8°C. The patient was unable to protect her airway and was moved to the medical intensive care unit following elective endotracheal intubation in the ward.At this point it was observed that the platelet count had progressively dropped since admission (Figure 3). It was 88,000/μL (ref. range: 150,000-450,000) by day 5. Changes in red cell concentration and haemoglobin concentration at this time are shown in Figure 4. Blood film taken on same day showed features of microangiopathic hemolysis (fragmented red cells) but coagulation parameters were unaltered; prothrombin time was 13.4 seconds (9.6-13.6), international normalized ratio (INR) was 1.2, activated partial thromboplastin time was 27 seconds (24-36), plasma fibrinogen level was 3.2 g/L (1.5-4.5) and D-dimer level was 0.4 mg/L (<0.2 mg/L). Direct and indirect Coombs’ tests were negative. Thromboelastometry results (clotting time, clot formation time, α angle and maximum clot firmness) also remained within normal limits despite thrombocytopenia. A possible explanation is that the pro-thrombotic state which exists in thrombotic microangiopathies negated the “tendency to bleed” due to thrombocytopenia.

Other investigation results at this point were as follows: serum aspartate aminotransferase (AST) 36 U/L (reference range: 10-35), alanine aminotransferase (ALT) 39 U/L (10-40), alkaline phosphatase 199 U/L (100-360), total bilirubin 38 μmol/L (5-21) (direct fraction 15%), serum ionized calcium 1.2 mmol/L (1-1.3), phosphorus 1.5 mmol/L (0.8-1.5), and magnesium 0.7 mmol/L (0.8-1.1). Blood and urine cultures were sterile. Non-contrast enhanced computed tomography of brain revealed no abnormalities. Magnetic resonance imaging of brain after gadolinium contrast revealed high T2 signal intensities in bilateral periventricular white matter without significant diffusion abnormalities. This was suggestive of white matter ischaemia or cerebral edema.

At the same time it was noted that the serum lactate dehydrogenase levels were significantly elevated (1660 IU/L, reference range 230-460). The overall picture suggested a diagnosis of thrombotic thrombocytopenic purpura (TTP). Despite this complication has never been reported following snakebite, we initiated therapeutic plasma exchange with fresh frozen plasma on day 7. Plasma exchange is the standard treatment for TTP and it was carried out at a frequency of one cycle every 48 hours. Following the third cycle of TPE, there was a gradual improvement in the level of consciousness, but the thrombocytopenia and features of microangiopathic hemolysis (MAHA) in blood film continued. After the seventh cycle of TPE there was a sustained rise in platelet count and features of MAHA gradually disappeared.

In the meantime, fourteen sessions of intermittent hemodialysis were carried out over six weeks, which led to a sustained improvement in renal functions. Her stay in the intensive care unit was also complicated by a ventilator-associated pneumonia and difficulty in weaning from the ventilator. With intense supportive care, intravenous antibiotics and chest physiotherapy, the patient made a full recovery and was discharged from the hospital on day 65. The platelet count picked up to 250,000/μL, lactate dehydrogenase level and other biochemical parameters such as liver and renal function tests were within normal limits at the point of discharge. She was clinically well and gradually returning to her daily routine at home two weeks later in the follow up clinic. She was subsequently discharged from our clinic to be followed up annually at the local hospital with regard to renal functions as cortical necrosis and chronic kidney disease, a potential complication of hump-nosed viper bites.

## Discussion

Adult TTP is an acute syndrome that comprises thrombocytopenia and microangiopathic hemolytic anemia associated with fever, varying degree of neurological dysfunction and renal impairment [5]. TTP exists in a spectrum with its counterpart hemolytic uremic syndrome (HUS). When the neurological impairment is a prominent manifestation, a diagnosis of TTP is favored, and if the renal impairment is prominent, HUS is favored [5]. Our patient had features of both these syndromes but it is not surprising as TTP and HUS are not mutually exclusive. They exist in a spectrum with either entity being at the two extremes. So, there can be patients with a mixed picture.

Most cases of TTP are associated with a deficiency of the metalloproteinase ADAMTS 13, which is responsible for cleavage of unusually large von Willebrand factor multimers in plasma. Its deficiency results in accumulation of these multimers giving rise to platelet aggregation, microthrombi formation and thrombotic microangiopathy [5]. Still, TTP can be observed in patients with normal ADAMTS 13 levels and a proposed hypotheses for pathogenesis in these circumstances include endothelial injury, increased platelet aggregation and increased levels of plasminogen-activator inhibitor type 1 (PAI-1). Acquired causes for adult TTP-HUS spectrum include bloody diarrheas associated with shiga toxin-producing *E. coli*, drugs (e.g. quinine, mitomycin, tacrolimus, sirolimus, cyclosporine), pregnancy and autoimmune diseases such as systemic lupus erythematosus. While primary or congenital cases of TTP are associated with a reduced level of ADAMTS 13, certain secondary causes of TTP may have normal levels of the enzyme [6,7].

Venom induced consumptive coagulation (VICC) is a well-known consequence of viper bites [8]. It is also known that some viper venoms, such as those of hump-nosed viper and Russell’s viper, can precipitate renal failure. The common tendency is to view both these manifestations as a part of a same syndrome. VICC is characterized by rapid onset coagulopathy within hours after the snake bite with elevated D-dimer levels, prolonged prothrombin time, and low fibrinogen levels which at times is associated with thrombocytopenia [9]. This resolves within 24 to 48 hours. It is not associated with systemic micro-thrombi and end-organ failure [9]. In two series of hump-nosed viper bites in Sri Lanka, coagulopathy was observed in only 21-39% of the sample [3,10]. Current evidence suggests that VICC and mechanisms of end organ damage – such as renal failure – are mutually exclusive though they can co-exist. It also follows that one can occur in absence of the other. That will explain the situation with our patient in which end organ failure was observed without any evidence of VICC.

This leads us to the question of the actual pathophysiology of end organ damage (which is renal failure in most occasions) in hump-nosed viper bites. It is thought to be due to venom induced thrombotic microangiopathy (TMA) [4,11]. It has also been suggested that TMA follows VICC in a subset of patients implicating the same toxin mediated pathology for both conditions [9]. What is interesting with this picture is that TTP-HUS spectrum is also characterized by thrombotic microangiopathy. The more frequent renal failure observed with hump-nosed viper bites is probably not often linked to HUS as there is no coagulopathy in this syndrome (which is frequently observed and mistakenly thought to be linked to renal failure in viper bites). However, if we follow the evidence that VICC is unrelated to organ failure and TMA can happen without VICC by a separate mechanism, then an acquired HUS-like syndrome will explain the clinical picture seen in patients with renal failure after a hump-nosed viper bite [12]. It also follows that if HUS-like manifestations may occur in a victim, TTP-like manifestations may also occur as a part of the same spectrum. Strangely, although renal failure is frequently reported secondary to microangiopathy (HUS equivalent), a TTP-like syndrome has never been reported. One reason may be that it is extremely rare to get a patient at the TTP end of the spectrum compared to the HUS end. Another reason may be that physicians confuse TMA and VICC (which may co-exist by independent mechanisms) to be a part of the same syndrome and misdiagnose the patient as having disseminated intravascular coagulation (DIC) [9]. So any TTP-like symptoms (confusion, seizures) may be attributed to DIC. This can result in inappropriate management strategies that can potentially harm the patient.

Though not exactly fitting to a diagnosis of TTP, syndromes suggestive of TTP-HUS spectrum has been reported in the literature with other venomous animal bites and stings [13-15]. Ho *et al.*[16] report an interesting case of tiger snake (*Notechis scutatus*) envenoming in which the patient had a somewhat similar course of illness to our patient. After an initial VICC, the patient had acute renal failure and a high LDH level with normal coagulation parameters. There was also microangiopathic hemolysis that followed resolution of VICC. The ADAMTS 13 levels, however, were normal and the patient did not have other clinical features of TTP. This patient received antivenom and also underwent plasmapheresis with subsequent recovery. However, the authors wondered, since ADAMTS 13 levels were normal, whether plasmapheresis was of any benefit. This observation has also been made by Casamento and Isbister [17] independently with tiger snake envenoming.

In our opinion, the data is not adequate to either confirm or refute the role of plasmapheresis in similar circumstances. Different envenoming may induce different patterns of clinical syndromes and it may be dangerous to generalize that in all or most snakebites ADAMTS 13 levels are normal. It is simply unknown and further data is necessary for each species. For example, envenoming by the scorpion *Hemiscorpius lepturus* is known to produce an HUS-like syndrome with low ADAMTS 13 levels and increased anti-ADAMTS 13 antibodies [14]. Furthermore, the clinical syndrome in our patient resembled more a classical picture of TTP than the patients cited in other case reports and there was no specific antivenom available. This justified our decision to go ahead with plasmapheresis based on the clinical picture, which resulted in a positive outcome. Unfortunately measuring ADAMTS 13 levels is a costly technique that is available in reference laboratories and currently not available to us in Sri Lanka. Given the increasing number of patients with acute renal failure and microangiopathy following *Hypnale* bites, data on ADAMTS 13 levels in *Hypnale* sp. envenoming should be a focus for further research.

### Limitations

As mentioned above we could not measure the ADAMTS 13 levels in this patient as the assay was unavailable. It is also possible that the renal failure in this patient occurred via an independent mechanism to TMA such as venom induced acute tubular necrosis. It is noted that the TTP-like syndrome (explainable by TMA) resolved rapidly with plasma exchange but the renal impairment took nearly six weeks to improve.

## Conclusions

Our patient is an excellent case to support the hypothesis that TMA in hump-nosed viper bites may occur in absence of VICC. Their origins may be related, but are independent. The manifestations of TMA favored a TTP-like syndrome rather than a HUS-like syndrome as she had all five classical features of TTP: fever, thrombocytopenia, microangiopathic hemolysis, renal impairment and neurological dysfunction (in the form of confusion and coma). Her clinical syndrome and relevant laboratory parameters improved after she was treated as for TTP. This unique case of a never before reported manifestation of hump-nosed viper envenoming encourages us to rethink about our current understanding of venom pathophysiology. The literature on this snake is sparse as bites are reported from a restricted geographical area (southern India and Sri Lanka). Our observations fit with the current thinking of venom toxinology and go one step further to show that a hitherto unknown, but theoretically plausible, life-threatening manifestation had actually occurred in real life. This case report also has an important message for clinicians to look out for a TTP-like syndrome in hump-nosed viper bites and not to confuse it with DIC. Appropriate timely treatment can be lifesaving.

## Consent

Written informed consent was obtained from the patient for publication of this case report.

## Competing interests

The authors declare that there are no competing interests.

## Authors’ contributions

All authors were involved in the management of patient. MW wrote the first draft. CR, AG and LG revised it. All authors have read and approved the final manuscript.

## Authors’ information

MW is a registrar in Medicine at the University Medical Unit of National Hospital of Sri Lanka. CR is lecturer in Medicine, Department of Clinical Medicine, Faculty of Medicine, University of Colombo. AG is a professor in Medicine at the Faculty of Medicine, University of Colombo. LG is a consultant hematologist and senior lecturer attached at the Department of Pathology, Faculty of Medicine, University of Colombo.
